# Mechanisms on Boron-Induced Alleviation of Aluminum-Toxicity in *Citrus grandis* Seedlings at a Transcriptional Level Revealed by cDNA-AFLP Analysis

**DOI:** 10.1371/journal.pone.0115485

**Published:** 2015-03-06

**Authors:** Xin-Xing Zhou, Lin-Tong Yang, Yi-Ping Qi, Peng Guo, Li-Song Chen

**Affiliations:** 1 College of Resource and Environmental Science, Fujian Agriculture and Forestry University, Fuzhou 350002, China; 2 Institute of Horticultural Plant Physiology, Biochemistry and Molecular Biology, Fujian Agriculture and Forestry University, Fuzhou 350002, China; 3 Institute of Materia Medica, Fujian Academy of Medical Sciences, Fuzhou 350001, China; 4 The Higher Educational Key Laboratory of Fujian Province for Soil Ecosystem Health and Regulation, Fujian Agriculture and Forestry University, Fuzhou 350002, China; 5 Fujian Key Laboratory for Plant Molecular and Cell Biology, Fujian Agriculture and Forestry University, Fuzhou 350002, China; USDA-ARS-SRRC, UNITED STATES

## Abstract

The physiological and biochemical mechanisms on boron (B)-induced alleviation of aluminum (B)-toxicity in plants have been examined in some details, but our understanding of the molecular mechanisms underlying these processes is very limited. In this study, we first used the cDNA-AFLP to investigate the gene expression patterns in *Citrus grandis* roots responsive to B and Al interactions, and isolated 100 differentially expressed genes. Results showed that genes related to detoxification of reactive oxygen species (ROS) and aldehydes (i.e., *glutathione S-transferase zeta class-like isoform X1*, *thioredoxin M-type 4*, and *2-alkenal reductase (NADP^+^-dependent)-like*), metabolism (i.e., *carboxylesterases and lecithin-cholesterol acyltransferase-like 4-like*, *nicotianamine aminotransferase A-like isoform X3*, *thiosulfate sulfurtransferase 18-like isoform X1*, and *FNR*, *root isozyme 2*), cell transport (i.e., *non-specific lipid-transfer protein-like protein At2g13820-like* and *major facilitator superfamily protein*), Ca signal and hormone (i.e., *calcium-binding protein CML19-like* and *IAA-amino acid hydrolase ILR1-like 4-like*), gene regulation (i.e., *Gag-pol polyprotein*) and cell wall modification (i.e., *glycosyl hydrolase family 10 protein*) might play a role in B-induced alleviation of Al-toxicity. Our results are useful not only for our understanding of molecular processes associated with B-induced alleviation of Al-toxicity, but also for obtaining key molecular genes to enhance Al-tolerance of plants in the future.

## Introduction

Aluminum (Al) is the most abundant metal and the third abundant element in earth’s crust after oxygen and silicon [1]. Al-toxicity is a major limiting factor for crop production in many acidic soils throughout the tropics and subtropics. Al-toxicity can inhibit the root growth which is the primary symptom of Al injury [2] through inhibiting root cell expansion and elongation.

Boron (B), as an essential element required for normal growth and development of higher plants, is absorbed from soil solution by plant roots mainly in the form of boron acid. B can alleviate Al-toxicity in many plants including lisianthus (*Eustoma grandiflorum*) [3], squash (*Cucurbita pepo*) [4], alfalfa (*Medicago sativa*) [5], *Citrus grandis* [6], flax (*Linum usitatissimum*) [7], pea (*Pisum sativum* [8], common bean (*Phaseolus vulgari*s) [9], sunflower (*Helianthus annuus*) [10], soybean (*Glycine max*) [11], apple (*Malus* sp.) rootstocks [12], cucumber (*Cucumis sativus*), maize (*Zea mays*) [13] and wheat (*Triticum aestivum*) [14].

B-deficiency is a widespread problem in many agricultural crops, including citrus [15]. Like Al-toxicity, B-deficiency also primarily inhibits root growth through limiting cell elongation rather than cell division [16]. In addition, Al is likely to be present as Al(OH)_3_, which is structurally similar to B(OH)_3_ [2]. Previous study showed that B-deficiency- or Al-toxicity-induced inhibition of root growth in squash plants could be a consequence of an impaired ascorbate (ASA) metabolism [17]. Based on the similarities of the molecules and of the symptom characteristic for Al-toxic and B-deficient plants, Blevins and Lukaszewski [18] proposed that Al-toxicity might exert its toxic effect by inducing B-deficiency. However, our studies with *C*. *grandis* seedlings showed that Al-toxicity increased or did not affect B concentration of roots, stems and leaves, demonstrating that the Al-induced growth inhibition was not caused by Al-induced B-deficiency [6]. It has been known that the primary function of B is related to the formation of primary cell walls, where it cross-links with the pectic polypectic polysaccharide rhamnogalacturonan II (RG-II). A higher degree of cross-linked RGII may contribute to a more stable network of cell walls with reduced pore sizes [19], thus preventing Al from getting into contact with sensitive targets at the plasma membrane and/or symplasm [13]. In addition, it has been suggested that B reduces the binding sites for Al in cell walls, thus ameliorating Al-toxicity [8,9]. Jiang et al. [6] showed that the antagonistic actions of B against inhibitory effects of Al-toxicity on *C*. *grandis* root growth was probably due to Al-induced alteration in Al speciation and/or sub-cellular compartmentation, and that B-induced alleviation of shoot and photosynthesis could be due to less accumulation in shoots. Corrales et al. [13] observed that B mitigated Al-induced damage of cell integrity in root tips, possibly through stimulating antioxidant responses in Al-stressed roots. Ruiz et al. [10] suggested that glutathione metabolism was one of the key processes for Al detoxification in sunflower. Recent study with flax showed that B decreased root activities of enzymes (i.e., phenylalanine ammonia-lyase, polyphenol oxidase and peroxidase) involved in phenolic compounds, and root concentrations of lignin and wall-bound phenols under Al-stress, thereby ameliorating Al-toxicity [7]. To conclude, the physiological and biochemical mechanisms on B-induced alleviation of Al-toxicity in plants have been examined in some details, our understanding of the molecular mechanisms underlying these processes is very limited.

Gene expression analyses offer us the opportunity to understand the molecular mechanisms involved in B-induced alleviation of plant Al-toxicity. Extensive research has shown that Al-toxicity affects the transcript levels of root genes associated with organic acid (OA) metabolism, OA transport and secretion, glycolytic pathways, carbohydrate and energy metabolism, cell wall modification, oxidative stress, protein metabolism, immobilization of Al by phosphate, signaling and hormones, gene regulation, cell death and senescence, and stress response [20–29]. Also, the effects of B-deficiency on root gene expression have been investigated by some workers [30–32]. However, very limited data are available on the differential expression of genes in response to B and Al interactions in plants.

Citrus belong to evergreen subtropical fruit trees cultivated in humid and subhumid tropical, subtropical and temperate regions of the world mainly on acidic soils. In China, high Al and low B are common in citrus plantations [6,33]. Although we investigated the effects of B and Al on citrus growth, the concentrations of B and Al in roots, stems and leaves, root and leaf OA metabolism, leaf photosynthesis and photosystem II photochemistry [6,34], there is hardly any information on the changes in gene expression of citrus roots in response to B and Al interactions. In this study, we investigated the effects of B and Al interactions on *C*. *grandis* growth, B and Al concentration in roots, and expression of root genes revealed by cDNA-amplified fragment length polymorphism (cDNA-AFLP). The objectives of this study were to understand the molecular mechanisms on B-induced alleviation of Al-toxicity in plants and to identify differentially expressed genes, which might contribute to B-induced alleviation of Al-toxicity.

## Materials and Methods

### Plant culture, B and Al treatments and sampling

This study was conducted from February to December, 2012 at Fujian Agriculture and Forestry University (FAFU), Fuzhou, China. Plant culture, treatments and sampling were performed according to Jiang et al. [6]. Briefly, 5-week-old seedlings of ‘Sour pummelo’ [*Citrus grandis* (L.) Osbeck] were transplanted to a 6 L pots (two plants per pot) containing fine river sand and grown in a greenhouse under natural photoperiod at FAFU. Six weeks after transplanting, seedlings were supplied with nutrient solution containing two B (i.e., 2.5 and 20 μM H_3_BO_3_) × two Al [i.e., 0 (-Al) and 1.2 mM AlCl_3_·6 H_2_O (+Al)] levels. The nutrient solution was formulated with macronutrients (in mM): KNO_3_, 1; Ca(NO_3_)_2_,1; KH_2_PO_4_, 0.1; and MgSO_4_, 0.5; and micronutrients (in μM): MnCl_2_, 2; ZnSO_4_, 2; CuSO_4_, 0.5; (NH_4_)_6_Mo_7_O_24_, 0.065; and Fe-EDTA, 20. The pH of the nutrient solution was adjusted to 4.1–4.2 using HCl or NaOH solution. There were 20 pots per treatment in a completely randomized design. Eighteen weeks after the beginning of B and Al treatments, approx. 5-mm-long root apices from new white roots were excised, immediately frozen in liquid N_2_ and stored at −80°C until extraction. The remaining seedlings that were not sampled were used to measure dry weight (DW), B and Al concentrations in roots.

### Plant DW, B and Al concentrations in roots

Ten plants per treatment from different replications were harvested and divided into their parts (shoots and roots). The plant parts were dried at 70°C for 48 h and DW were measured.

For the determination of B and Al, fibrous roots were collected and dried. B was assayed by the modified curcumin method [35] after samples were ashed at 500°C for 5 h, and dissolved in 0.1 M HCl. Al was assayed by the aluminon method [36] after samples were digested in a mixture of HNO_3_: HClO_4_ (5:1 v/v).

### Collection of root exudates and determination of malate and citrate in exudates

Root exudates were collected according to Yang et al. [37]. Briefly, 18 weeks after the beginning of B and Al treatments, ten to twelve approx. 5-mm-long root apices from new white roots were excised, then collected in Petri dishes containing 5 mL control solution (0.5 mM CaCl_2_, pH 4.1–4.2). After three rinses with 5 mL control solution (each for 20 min), the root apices were transferred to 2 mL centrifuge tubes containing 1 mL control solution in the absence or presence of 0.5 mM AlCl_3_·6H_2_O (pH 4.1–4.2). The tubes were placed vertically on a shaker (200 rpm) at dark. The treatment times for malate and citrate collection were 12 and 24 h, respectively. Malate and citrate in exudates were assayed by enzymatic method [37].

### RNA extraction, cDNA synthesis and cDNA-AFLP analysis

Root tips of six plants from different pots were mixed as a biological replicate. Equal amounts of root tips were collected from each plant. There were three biological replicates for each treatment (total of 18 plants from 18 pots). Total RNA were independently extracted three times from four B and Al combinations using Recalcirtant Plant Total RNA Extraction Kit (Centrifugal column type, Bioteke Corporation, China) according to manufacturer’s instructions. cDNA synthesis and cDNA-AFLP analysis were performed according to Zhou et al. [38].

### Quantitative RT-PCR (qRT-PCR) analysis

Total RNA extracted as described above was used for qRT-PCR analysis, which was performed according to Zhou et al. [38]. The primers of candidate TDFs were listed in S1 Table.

### Experimental design and statistical analysis

There were 20 pots (40 seedlings) per treatment in a completely randomized design. Experiments were performed with 3–10 replicates. Results represented the means ± SE. Differences among four treatments were analyzed by two × two ANOVA. Means were separated by the Duncan's new multiple range test at *P* < 0.05 level.

## Results

### Effects of B and Al interactions on seedling growth, Al and B concentrations in roots

**Fig 1:**
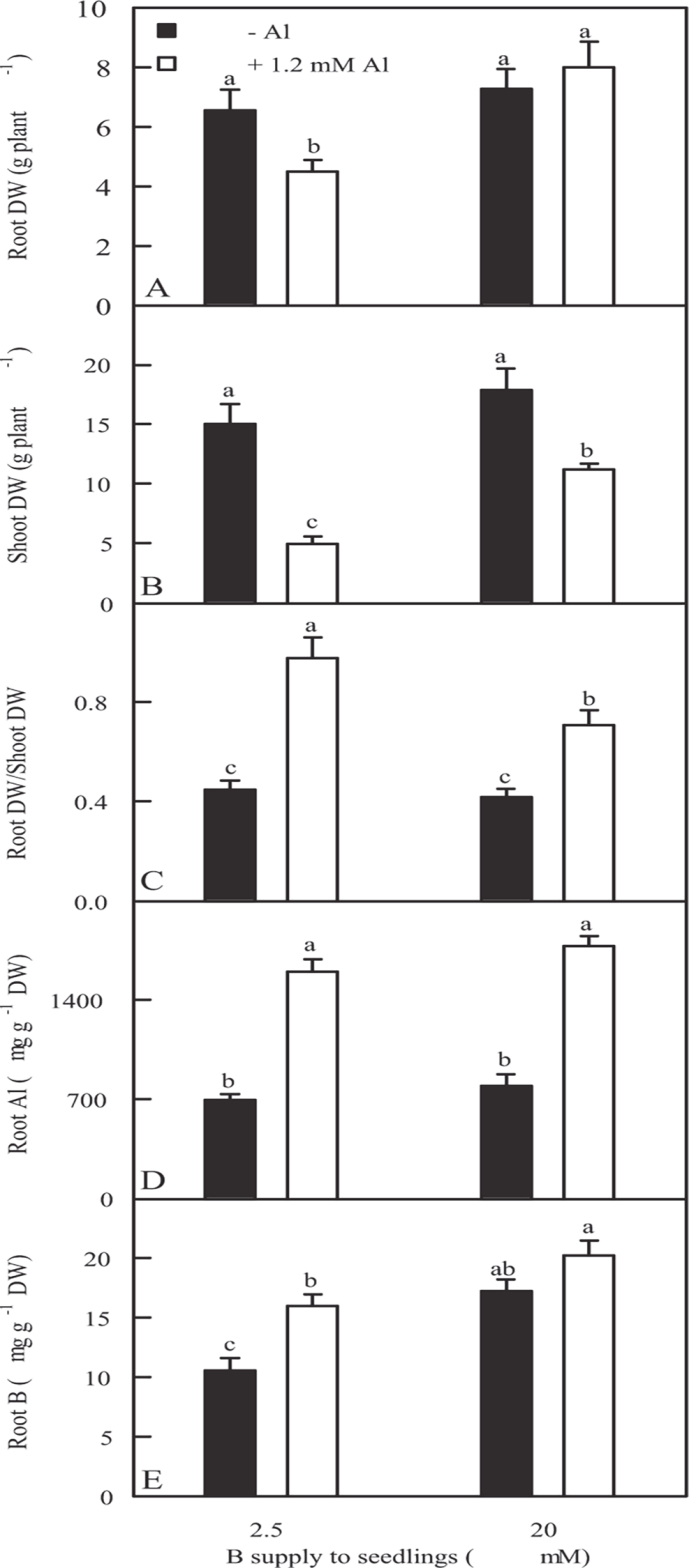
Effects of B-Al interactions on root DW (A), shoot DW (B), root DW/shoot DW ratio (C), root Al (D) and B (E) concentrations in *C*. *grandis* seedlings. Data are means ± SE (*n* = 10 except for 5 for root Al and B concentrations DW). Differences among four treatments were analyzed by 2 (B levels) × 2 (Al levels) ANOVA. Different letters indicate a significant difference at *P* < 0.05.

In non-Al-treated (-Al) seedlings, root DW, shoot DW and root DW/shoot DW ratio did not significantly change in response to B supply. In Al-treated (+Al) seedlings, both root DW and shoot DW were higher under 20 μM B than under 2.5μM B, while root DW/shoot DW ratio was lower under 20 μM B. Al decreased root DW and shoot DW except for a similar root DW between Al treatments under 20 μM B, and increased root DW/shoot DW ratio (Fig. 1A-C).

Al increased root Al concentration, whereas B did not significantly affect root Al concentration (Fig. 1D). B supply increased root B concentration. B concentration was higher in +Al roots than in −Al roots under 2.5 μM B, while B concentration in 20 μM B-treated roots did not differ between the two Al treatments (Fig. 1E).

### Effects of B and Al interactions on Al-induced secretion of malate and citrate from roots

**Fig 2:**
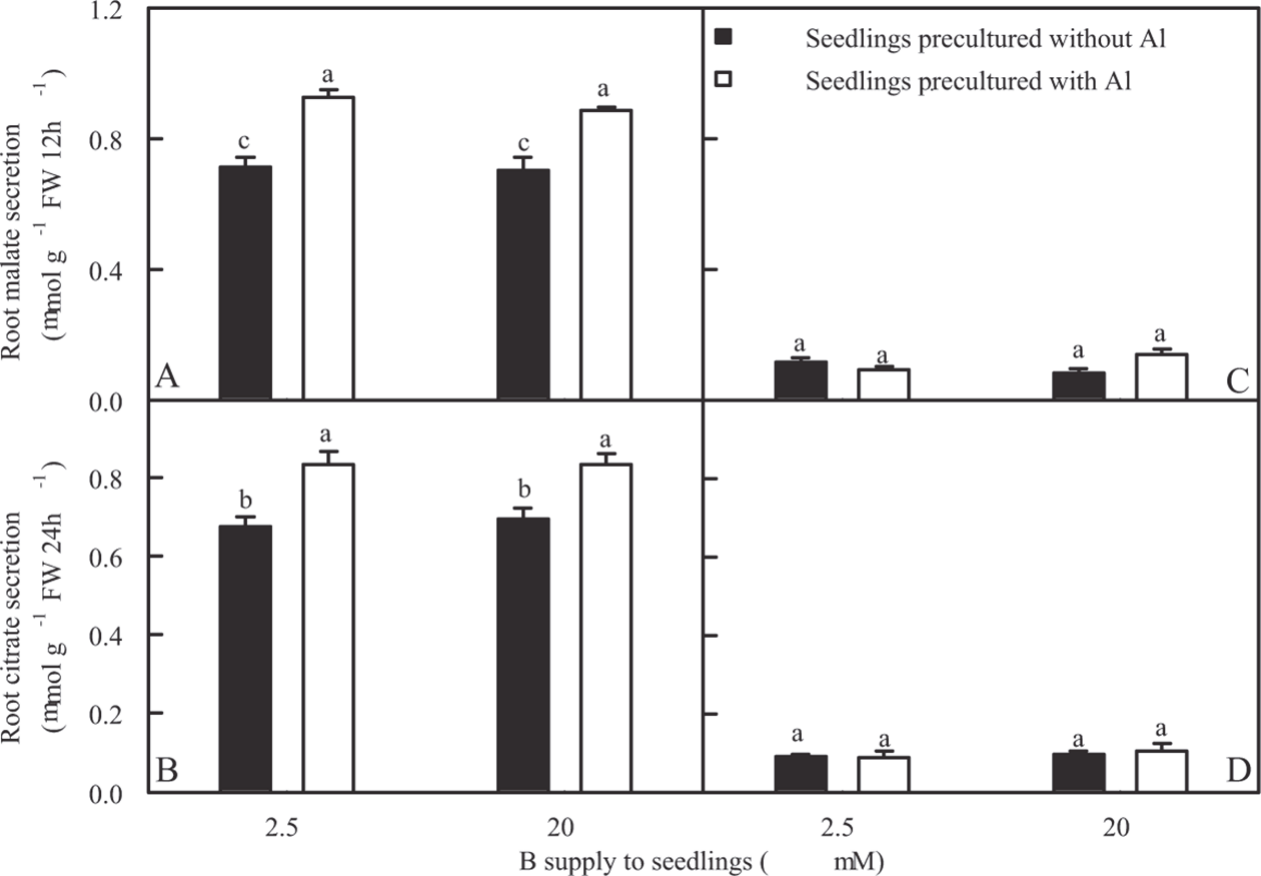
Al-induced-secretion of malate (A and C) and citrate (B and D) by excised from *C*. *grandis* seedlings treated with different B and Al levels. Malate and citrate secretion from excised roots were measured after 12 or 24 h treatment, respectively in 0.5 mM CaCl_2_ + 0.5 mM AlCl_3_·6H_2_O (A and B) or 0.5 mM CaCl_2_ solution (C and D), pH 4.1–4.2. Bars represent means ± SE (*n* = 4). Differences among four treatments were analyzed by 2 (B levels) × 2 (Al levels) ANOVA. Different letters indicate a significant difference at *P* < 0.05.

B supply did not significantly affect Al-induced secretion of malate and citrate from +Al excised or −Al excised roots. Al-induced secretion of malate and citrate from +Al excised roots was higher than from −Al excised roots (Fig. 2).

### Identification of root differentially expressed genes and their expression patterns under B-Al interactions

We used a total of 256 selective primer combinations for cDNA-AFLP analysis in order to isolate the differentially expressed transcript-derived fragments (TDFs) responsive to B and Al interactions. In this study, approx. 5970 clear and unambiguous TDFs were amplified, with an average of 29.5 (7–52) TDFs for each primer combination. A total of 169 differentially expressed and reproducible TDFs were obtained. All these TDFs were reamplified, cloned and sequenced, and 142 cDNA fragments produced useable sequence data. Homology analyses were conducted using BLAST from GenBank. Among these TDFs, 89 TDFs showed significant homology to genes encoding known or putative proteins; 11 TDFs were homologus to genes encoding uncharacterized and hypothetical proteins; and the remaining 42 TDFs did not show homologus to any nucleotide or amino sequence in the public databases. These TDFs were associated with metabolism (21), stress response (10), autophagy and senescence (15), signal transduction and hormone (12), gene regulation (15), cell transport (12), cell wall modification (4) and others (11). Further analysis showed that in 2.5 (20) μM B-treated roots, 25 (35) TDFs were upregulated by Al-toxicity, and 36 (29) TDFs were downregulated by Al-toxicity; and in −Al (+Al) roots, the expression levels of 22 (30) TDFs increased and 36 (22) TDFs decreased as B supply increased from 2.5 to 20 μM. Obviously, B-Al interaction affected root gene expression (Tables 1 and 2).

**Table 1:** Homology of differentially expressed cDNA-AFLP fragments with known gene sequences in database using BLASTN algorithm along their expression patterns in roots from *Citrus grandis* seedlings treated with two B × Al levels.

TDF#	Genebank ID	E value	Max score	Organism origin	Size (bp)	Description	Identity	Fold change			
								2.5 B -Al	2.5 B + Al	20 B - Al	20 B + Al
***Metabolism***											
157-6	XP_006479398	2.E-30	118	*Citrus sinensis*	185	Flavonol synthase/flavanone 3-hydroxylase-like	100%	0 b	1.00 a	0 b	1.03 a
134-14	NP_197540	5.E-15	76.3	*Arabidopsis thaliana*	171	Flavanone 3 hydroxylase-like protein	65%	1.00 b	2.80 a	0.07 c	0.21 c
149-2	XP_006487080	7.E-29	115	*Citrus sinensis*	255	Probable carboxylesterase 12-like	81%	1.00 b	1.08 ab	0.12 c	1.22 a
216-2	XP_006490283	3.E-41	147	*Citrus sinensis*	236	Carboxylesterase 1-like	95%	1.00 b	0.16 c	0.16 c	1.47 a
250-3	XP_006468458	4.E-51	179	*Citrus sinensis*	297	Lecithin-cholesterol acyltransferase-like 4-like	99%	1.00 a	0.45 b	1.10 a	1.01 a
51-12	YP_740484	9.E-24	101	*Citrus sinensis*	162	Acetyl-CoA carboxylase carboxyltransferase beta subunit	98%	1.00 a	0.05 b	0.05 b	0.05 b
136-3	XP_006492541	2.E-39	145	*Citrus sinensis*	239	Adenosylhomocysteinase-like	91%	1.00 b	1.06 b	0.10 c	5.97 a
141-5	XP_006471128	6.E-50	172	*Citrus sinensis*	279	Probable S-adenosylmethionine-dependent methyltransferase At5g37990-like	95%	1.00 a	0.08 b	0.09 b	0.08 b
87-2	NP_180524	8.E-33	129	*Arabidopsis thaliana*	279	Phosphomethylpyrimidine synthase	76%	1.00 a	0.10 b	0.11 b	0.10 b
138-5	XP_006469907	5.E-20	90.9	*Citrus sinensis*	256	Nicotianamine aminotransferase A-like isoform X3	75%	0 b	1.00 a	0 b	1.02 a
138-3	NP_567934	4.E-44	158	*Arabidopsis thaliana*	276	LL-diaminopimelate aminotransferase	83%	1.00 a	1.05 a	1.06 a	0.13 b
134-12	XP_007043658	2.E-24	103	*Theobroma cacao*	256	Tyrosine transaminase family protein	80%	1.00 a	1.08 a	1.10 a	0.06 b
18-2	XP_006466965	1.E-41	143	*Citrus sinensis*	225	Thiosulfate sulfurtransferase 18-like isoform X1	92%	1.00 a	0.16 b	0.17 b	1.07 a
178-4	XP_006466965	9.E-41	141	*Citrus sinensis*	225	Thiosulfate sulfurtransferase 18-like isoform X1	92%	1.00 a	0.22 b	0.18 b	1.03 a
54-2	XP_002308954	2.E-24	101	*Populus trichocarpa*	189	40S ribosomal protein S2	84%	1.00 c	15.58 a	4.60 b	15.11 a
80-2	XP_003523292	2.E-56	184	*Glycine max*	313	60S ribosomal protein L10	95%	1.00 b	1.03 b	6.49 a	1.09 b
201-1	NP_564355	1.E-30	119	*Arabidopsis thaliana*	201	Ferredoxin-NADP reductase, root isozyme 2	90%	1.00 a	1.03 a	0.16 b	1.09 a
25-4	ACG28186	3.E-32	121	*Zea mays*	212	Cytochrome b6-f complex iron-sulfur subunit	85%	1.00 a	0.18 b	0.08 b	0.09 b
29-2	ACG28186	5.E-31	118	*Zea mays*	212	Cytochrome b6-f complex iron-sulfur subunit	84%	1.00 a	0.27 b	0.24 b	0.21 b
134-9	XP_002518810	3.E-18	87.8	*Ricinus communis*	232	Electron transporter, putative	75%	1.00 b	1.02 b	1.13 b	21.82 a
51-9	XP_002531030	2.E-16	76.3	*Ricinus communis*	175	Ribulose-bisphosphate carboxylase, putative	94%	1.00 a	0.30 b	0.32 b	0.04 c
***Stress response***											
78-4	XP_006470782	8.E-12	67	*Citrus sinensis*	250	Glutathione S-transferase zeta class-like isoform X1	100%	1.00 a	0.26 b	0.24 b	0.97 a
164-1	XP_006493708	3.E-04	47.8	*Citrus sinensis*	299	Glutathione reductase, cytosolic-like	88%	1.00 a	1.03 a	0.14 b	1.02 a
217-2	NP_192897	9.E-14	72.4	*Arabidopsis thaliana*	227	Glutathione peroxidase 6	63%	1.00 b	1.23 b	9.45 a	1.20 b
60-1	XP_007021413	4.E-10	62.4	*Theobroma cacao*	300	Thioredoxin M-type 4	71%	1.00 b	1.07 b	1.15 b	10.70 a
243-1	XP_006475833	1.E-38	141	*Citrus sinensis*	242	2-alkenal reductase (NADP^+^- dependent) -like	97%	1.00 b	6.00 a	1.06 b	6.15 a
178-1	XP_007017815	5.E-05	48.1	*Theobroma cacao*	304	Chaperone DnaJ-domain superfamily protein, putative	70%	1.00 a	0.14 c	0.39 b	0.39 b
83-5	BAJ11779	5.E+00	30.8	*Corchorus tridens*	125	Dehydration responsive protein	78%	1.00 c	9.62 ab	1.34 bc	9.92 a
219-3	XP_007035783	1.E+00	35.4	*Theobroma cacao*	212	Adenine nucleotide alpha hydrolases-like superfamily protein	84%	1.00 b	7.91 a	1.02 b	5.71 a
59-1	XP_002310744	1.E-13	75.9	*Populus trichocarpa*	282	Disease resistance family protein	41%	1.00 a	0.08 b	0.25 b	0.09 b
176-1	XP_006494011	2.E-21	98.2	*Citrus sinensis*	286	Putative disease resistance protein At3g14460-like	66%	1.00 a	1.09 a	0.23 b	1.02 a
***Autophagy and senescence***											
158-1	NP_564664	4.E-11	67.8	*Arabidopsis thaliana*	236	Autophagy 18H-like protein	53%	1.00 b	0.23 c	2.69 a	0.23 c
2-1	BAB33421	8.E-39	140	*Pisum sativum*	244	Putative senescence-associated protein	86%	1.00 a	1.04 a	1.06 a	0.12 b
5-3	BAB33421	1.E-25	104	*Pisum sativum*	191	Putative senescence-associated protein	89%	1.00 a	0.96 a	1.02 a	0.15 b
139-8	BAB33421	2.E-35	131	*Pisum sativum*	236	Putative senescence-associated protein	89%	1.00 b	0 c	0 c	3.62 a
156-3	BAB33421	3.E-08	58.2	*Pisum sativum*	274	Putative senescence-associated protein	69%	1.00 a	0.17 b	1.06 a	1.06 a
141-7	AAR25995	3.E-31	115	*Pyrus communis*	259	Putative senescence-associated protein	96%	1.00 b	1.01 b	1.04 b	7.99 a
209-1	AAR25995	1.E-56	181	*Pyrus communis*	296	Putative senescence-associated protein	98%	1.00 b	8.39 a	1.18 b	0.95 b
217-1	AAR25995	2.E-51	167	*Pyrus communis*	309	Putative senescence-associated protein	99%	1.00 b	3.31 a	2.86 ab	1.14 b
219-2	AAR25995	2.E-50	165	*Pyrus communis*	296	Putative senescence-associated protein	97%	1.00 b	1.14 b	10.80 a	1.11 b
223-1	AAR25995	2.E-49	162	*Pyrus communis*	314	Putative senescence-associated protein	97%	1.00 a	0.16 b	1.09 a	0.99 a
179-6	XP_006473584	1.E-37	138	*Citrus sinensis*	215	Cysteine proteinase 15A-like	98%	0 c	0 c	1.00 a	0.54 b
246-9	XP_006467009	2.E-21	94.7	*Citrus sinensis*	154	Aspartic proteinase-like protein 1-like	94%	1.00 b	1.06 b	5.68 a	1.13 b
87-3	XP_002882118	7.E-34	133	*Arabidopsis lyrata subsp*. *lyrata*	272	Serine-type peptidase	71%	1.00 b	1.16 b	4.32 a	1.25 b
179-4	XP_003633155	3.E-19	90.5	*Vitis vinifera*	238	Ubiquitin carboxyl-terminal hydrolase 22-like	93%	1.00 a	0.05 c	0.06 c	0.48 b
78-2	XP_006484457	1.E-29	117	*Citrus sinensis*	208	Ubiquitin receptor RAD23c-like	98%	1.00 b	1.16 b	5.76 a	1.07 b
***Signal transduction and hormone***											
19-4	XP_003549848	2.E-25	102	*Glycine max*	274	Putative calcium-binding protein CML19-like	63%	1.00 b	0.99 b	3.68 ab	4.15 a
19-5	XP_003549848	2.E-25	102	*Glycine max*	274	Putative calcium-binding protein CML19-like	63%	1.00 c	1.42 bc	4.75 a	4.43 ab
89-2	NP_178383	1.E-43	156	*Arabidopsis thaliana*	258	Protein kinase 2B	88%	1.00 a	0.15 b	1.11 a	1.26 a
25-3	CAB63149	3.E-36	136	*Arabidopsis thaliana*	222	MAP kinase	92%	1.00 a	0.24 b	1.08 a	0.25 b
140-2	XP_006485632	6.E-07	54.7	*Citrus sinensis*	219	Probable receptor-like protein kinase At5g47070-like isoform X1	96%	1.00 a	1.06 a	1.08 a	0.35 b
246-3	XP_003534233	7.E-03	42.7	*Glycine max*	221	SRSF protein kinase 1-like isoform 1	71%	1.00 a	0.05 b	0.05 b	0.06 b
51-15	CAB90633	2.E-05	48.9	*Fagus sylvatica*	116	protein phopsphatase 2C (PP2C)	81%	1.00 a	0.21 b	0.23 b	0.24 b
131-1	XP_006350060	2.E-07	55.8	*Solanum tuberosum*	288	Tetraspanin-8-like	66%	1.00 a	0.05 b	0.06 b	0.05 b
138-6	NP_973890	1.E-11	67.8	*Arabidopsis thaliana*	237	COP9 signalosome complex subunit 5a	89%	1.00 c	1.80 b	2.53 a	0.24 d
141-9	XP_006476047	6.E-12	68.9	*Citrus sinensis*	183	Ankyrin repeat-containing protein At3g12360-like	79%	1.00 a	0.33 b	0.35 b	0.35 b
87-7	XP_006468682	2.E-25	107	*Citrus sinensis*	232	WD repeat-containing protein 26-like isoform X1	98%	1.00 b	12.03 a	1.70 b	2.06 b
178-5	XP_006475371	4.E-19	87.8	*Citrus sinensis*	149	IAA-amino acid hydrolase ILR1-like 4-like	98%	0	0	0	+
***Gene regulation***											
188-3	ADL36732	4.E-09	60.5	*Malus domestica*	228	HSF domain class transcription factor	53%	1.00 a	1.09 a	0.28 b	1.01 a
23-1	XP_006466606	3.E-30	117	*Citrus sinensis*	198	Heat shock factor protein HSF24-like	97%	1.00 b	3.36 a	3.27 a	3.20 a
138-4	XP_007018496	4.E-03	44.3	*Theobroma cacao*	261	PHD finger transcription factor	44%	0	0	+	0
139-1	XP_006468886	5.E-74	246	*Citrus sinensis*	375	Putative pentatricopeptide repeat-containing protein At2g01510-like	98%	1.00 a	0.57 b	0.57 b	1.11 a
177-3	NP_195386	4.E-05	49.7	*Arabidopsis thaliana*	235	Pentatricopeptide repeat-containing protein	73%	0	+	0	0
27-4	XP_006467029	7.E-27	112	*Citrus sinensis*	186	DNA-directed RNA polymerase II subunit 1-like isoform X3	98%	1.00 a	0.14 b	1.04 a	0.97 a
132-1	XP_006479511	2.E-39	149	*Citrus sinensis*	245	DNA repair and recombination protein RAD26-like isoform X3	99%	0	0	0	+
219-4	XP_006472001	3.E-15	76.6	*Citrus sinensis*	189	DNA excision repair protein ERCC-1-like isoform X1	84%	1.00 b	11.60 a	1.16 b	1.07 b
177-8	XP_006490371	1.E-10	65.5	*Citrus sinensis*	165	DNA mismatch repair protein MSH3-like	97%	1.00 a	1.02 a	0.22 b	0.21 b
134-13	BAK61840	2.E-16	82.8	*Citrus unshiu*	182	Gag-pol polyprotein	70%	1.00 b	0.04 c	0.04 c	3.25 a
134-4	XP_003614387	3.E-14	77.4	*Medicago truncatula*	279	RRNA intron-encoded homing endonuclease	93%	+	0	0	0
246-2	XP_003614387	1.E-08	60.5	*Medicago truncatula*	234	RRNA intron-encoded homing endonuclease	86%	1.00 b	10.50 a	1.39 b	1.05 b
83-2	XP_003614389	3.E-22	97.1	*Medicago truncatula*	210	RRNA intron-encoded homing endonuclease	87%	1.00 a	0.13 b	1.13 a	0.12 b
162-5	XP_006473637	3.E-07	55.1	*Citrus sinensis*	149	5'-3' exoribonuclease 3-like isoform X2	96%	0	+	0	0
246-5	XP_006472153	9.E-24	100	*Citrus sinensis*	169	Pre-mRNA-splicing factor 38A-like	94%	1.00 b	1.09 b	10.40 a	1.09 b
***Cell transport***											
175-7	XP_006489422.	3.E-06	49.7	*Citrus sinensis*	136	Non-specific lipid-transfer protein-like protein At2g13820-like	96%	0 c	0 c	1.00 a	0.38 b
59-3	XP_007026766	7.E+00	33.9	*Theobroma cacao*	243	Major facilitator superfamily protein, putative	32%	0 c	0 c	1.00 a	0.23 b
252-1	XP_006464865	4.E-34	126	*Citrus sinensis*	275	Citrate-binding protein-like	77%	0 c	1.00 a	0 c	0.35 b
141-8	XP_006473247	3.E-31	123	*Citrus sinensis*	209	Patellin-2-like	84%	0	0	0	+
177-6	XP_007012650	2.E-03	43.5	*Theobroma cacao*	202	Membrane lipoprotein	82%	0	0	0	+
134-5	XP_006469059	2.E-35	130	*Citrus sinensis*	260	Ras-related protein RABA1f-like	95%	1.00 b	3.37 a	0.16 c	2.79 a
19-3	XP_006467607	3.E-57	194	*Citrus sinensis*	316	Protein transport protein Sec61 subunit alpha-like	94%	1.00 b	7.14 a	6.69 a	6.77 a
180-2	XP_006480618	2.E-11	65.9	*Citrus sinensis*	240	Syntaxin-71-like	79%	1.00 a	0.06 b	1.14 a	0.05 b
162-4	XP_006487552	1.E-15	80.1	*Citrus sinensis*	177	ADP-ribosylation factor GTPase-activating protein AGD3-like	100%	0	+	0	0
78-3	XP_006483372	3.E-11	66.2	*Citrus sinensis*	147	Putative clathrin assembly protein At2g25430-like	97%	1.00 a	1.02 a	1.06 a	0.10 b
136-8	XP_006472885	2.E-29	117	*Citrus sinensis*	200	Target of Myb protein 1-like isoform X1	92%	0	0	+	0
87-5	AFX72760	5.E-32	124	*Litchi chinensis*	236	ATP/ADP carrier protein, partial	97%	1.00 a	0.16 b	0.10 b	0.13 b
***Cell wall modification***											
124-1	XP_006480190	1.E-12	70.1	*Citrus sinensis*	149	Probable pectate lyase 8-like	94%	1.00 a	0.08 b	1.03 a	0.07 b
17-1	XP_006493306	2.E-44	160	*Citrus sinensis*	257	Probable pectinesterase/pectinesterase inhibitor 61-like	97%	0	+	0	0
51-1	XP_007042653	4.E-25	107	*Theobroma cacao*	241	Glycosyl hydrolase family 10 protein, putative	65%	1.00 b	0.11 c	0.09 c	1.64 a
148-1	XP_006469451	6.E-01	36.6	*Citrus sinensis*	189	Fasciclin-like arabinogalactan protein 2-like	90%	1.00 a	1.08 a	0.17 b	0.20 b
***Others (unknown/unclassified)***											
204-2	XP_003588355	1.E-04	48.1	*Medicago truncatula*	170	Mitochondrial protein, putative	88%	1.00 a	0.59 b	1.09 a	0.57 b
149-1	XP_006480893	7.E-43	155	*Citrus sinensis*	276	Uncharacterized protein LOC102616798	70%	0	0	+	0
159-2	XP_006468400.	2.E-13	73.2	*Citrus sinensis*	219	Uncharacterized protein LOC102609810	78%	0 b	1.00 a	0 b	0.99 a
179-1	NP_001169009	2.E-23	98.6	*Zea mays*	318	Hypothetical protein	74%	0	0	0	+
180-5	XP_006421131	8.E-18	84.3	*Citrus clementina*	260	Hypothetical protein CICLE_v10005475mg	87%	1.00 a	0.06 b	0.07 b	0.07 b
187-2	XP_006492168	2.E-06	54.7	*Citrus sinensis*	292	Uncharacterized protein LOC102628400	87%	1.00 c	4.51 b	1.23 c	9.38 a
19-2	NP_189331	7.E-18	85.9	*Arabidopsis thaliana*	206	Uncharacterized protein	82%	1.00 a	0.15 b	0.18 b	0.16 b
237-1	XP_006478809	2.E-46	163	*Citrus sinensis*	263	Uncharacterized protein LOC102629577	100%	1.00 b	5.24 a	1.19 b	5.21 a
249-1	XP_004499954	2.E-37	131	*Cicer arietinum*	305	Uncharacterized protein LOC101515437	96%	1.00 b	5.93 a	6.66 a	1.01 b
87-4	XP_006444509	4.E-14	74.3	*Citrus clementina*	249	hypothetical protein CICLE_v10021318mg	97%	1.00 b	0.92 b	11.3 a	11.9 a
204-1	XP_003608262	1.E-09	60.5	*Medicago truncatula*	252	hypothetical protein MTR_4g091430	76%	1.00 a	1.14 a	0.57 b	1.16 a

*Note*. 2.5 B - Al: 2.5 μM B + 0 mM Al; 2.5 B + Al: 2.5 μM B + 1.2 mM Al; 20 B - Al: 20 μM B + 0 mM Al; 20 B + Al: 20 μM B + 1.2 mM Al.Ratio means the ratio of different treatments to control (set as 1). Usually, the control was 2.5 B - Al. If TDF was not detected in the treatment, the control would be 2.5 B + Al and so on.

0 means TDF was not detected in the treatment; + means TDF was detected only in the treatment.

Values are means of 3 replicates.

Differences among four treatments were analyzed by two (B) × two (Al) ANOVA.

Within a row, values followed by different letters indicate a significant difference at *P* < 0.05.

**Table 2:** Summary of differentially expressed TDFs in roots from *Citrus grandis* seedlings treated with two B (2.5 and 20 μM H_3_BO_3_) and two Al (0 and 1.2 mM AlCl_3_·6H_2_O) level.

	Total differentially expressed TDFs	Al-toxicity-responsive TDFs				20 μM B-responsive TDFs			
		2.5 μM B		20 μM B		0 mM Al		1.2 mM Al	
		Up	Down	Up	Down	Up	Down	Up	Down
Metabolism	21	4	10	10	4	2	13	5	4
Stress response	10	3	3	7	1	1	5	3	0
Autophagy and senescence	15	2	5	3	9	6	2	6	4
Signal transduction and hormone	12	2	6	1	3	3	4	4	3
Gene regulation	15	5	5	4	3	3	5	4	5
Cell transport	12	4	2	4	5	4	2	4	3
Cell wall modification	4	1	2	1	1	0	2	1	2
Others	11	4	3	5	3	3	3	3	1
Total	100	25	36	35	29	22	36	30	22

### Validation of cDNA-AFLP data

**Fig 3:**
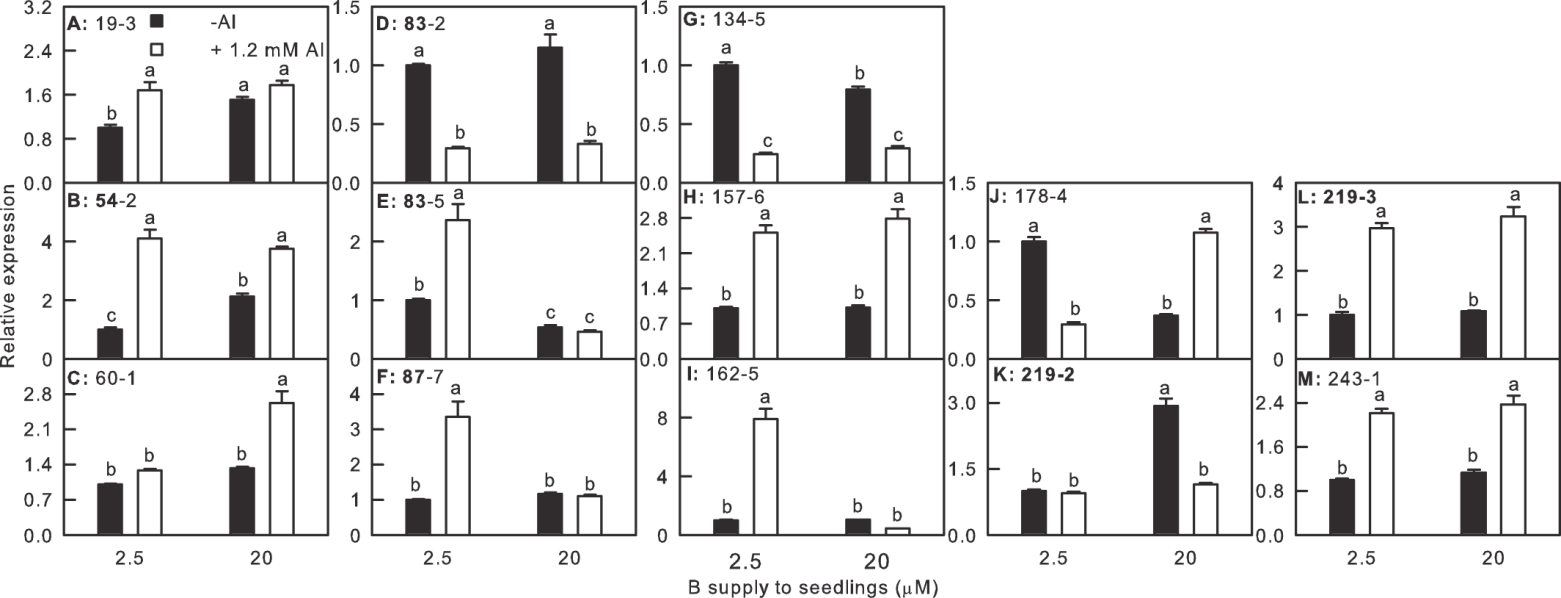
Relative expression levels of 13 genes in roots from *C*. *grandis seedlings* treated with different B and Al levels. (A) Protein transport protein Sec61 subunit alpha-like (TDF #19-3); (B) 40S ribosomal protein S2 (TDF #54-2); (C) Thioredoxin M-type 4 (TDF #60-1); (D) RRNA intron-encoded homing endonuclease (TDF #83-2); (E) Dehydration responsive protein (TDF #83-5); (F) WD repeat-containing protein 26-like isoform X1 (TDF #87-7); (G) Ras-related protein RABA1f-like (TDF #134-5); (H) Flavonol synthase/flavanone 3-hydroxylase-like (TDF #157-6); (I) 5'-3' exoribonuclease 3-like isoform X2 (TDF #162-5); (J) Thiosulfate sulfurtransferase 18-like isoform X1 (TDF #178-4); (K) Putative senescence-associated protein (TDF #219-2); (L) Adenine nucleotide alpha hydrolases-like superfamily protein (TDF #219-3) and (M) 2-alkenal reductase (NADP^+^- dependent)-like (TDF #243-1). Bars represent means ± SE (*n* = 4). Samples for qRT-PCR were run in at least three biological replicates with two technical replicates. Relative gene expression was calculated using ddCt algorithm. For the normalization of gene, citrus *actin* (GU911361.1) was used as an internal standard and the roots from 2.5 μM B + 0 mM Al-treated plants was used as reference sample, which was set to 1. Differences among four treatments were analyzed by 2 (B levels) × 2 (Al levels) ANOVA. Different letters indicate a significant difference at *P* < 0.05.

To validate the reliabiability of cDNA-AFLP expression patterns, 13 TDFs were selected for qRT-PCR analysis. Among these TDFs, 11 TDFs (i.e., TDFs #19-3, 54-2, 60-1, 83-2, 87-7, 157-6, 162-5, 178-4, 219-2, 219-3 and 243-1) matched well with the expression profiles observed with cDNA-AFLP (Fig. 3). This technique was thus validated in 84.7% of cases. In addition, a linear regression analysis between qRT-PCR results and cDNA-AFLP data was performed. The correlation coefficient (*r*) was 0.8501, demonstrating that the qPCR and cDNA-AFLP results were highly correlated (S2 Fig.). It is worth noting that *5'-3' exoribonuclease 3-like isoform X2* (TDF #162-5) was not included in the analysis because the TDF was detected only in 2.5 μM B + 1.2 mM Al-treated roots.

## Discussion

### B-induced amelioration of Al-toxicity in *C*. *grandis*

Our results showed that the effects of Al-toxicity on root DW, shoot DW and root DW/shoot DW ratio was less pronounced under 20 μM B than under 2.5 μM B (Fig. 1A-C), demonstrating that B alleviated Al-toxicity in *C*. *grandis* seedlings. Our data and previous study showed that Al-toxicity increased or did not affect B concentration in roots (Fig. 1E), stems and leaves [6], meaning that B-induced mitigation of Al-toxicity was not caused by an increase in plant B concentration, as previously obtained on *C*. *grandis* [6], flax [7] and soybean [39]. Al-induced secretion of OA anions from roots has been known to be a major mechanism of Al-tolerance in plants [40]. Our results showed that Al-induced secretion of malate and citrate from +Al or -Al excised roots was not affected by B supply (Fig. 2), indicating that B-induced alleviation of Al-toxicity was not explained in this way. Al-tolerance of plants is associated not only with low Al uptake, but also with relatively little Al translocation from roots to shoots [37,41]. In +Al seedlings, root Al concentration did not differ between two B treatments (Fig. 1D), while B supply decreased stem and leaf Al concentration [6], meaning that relatively less amount of Al was transported from roots to leaves (shoots). This might contribute to B-induced alleviation of Al-toxicity in *C*. *grandis* seedlings.

### Genes related to metabolism

Twenty one TDFs involved in metabolism were altered by B and Al interactions (Tables 1 and 2). Phenolic compounds particularly flavonoid type phenolics have been shown to confer plant Al-tolerance *via* the dual mechanisms of antioxidation and Al chelation [40]. Tolrà et al. [42] showed that root concentrations of caffeic acid, catechol and catechin were higher in Al-tolerant maize cultivar than in sensitive cultivar. Our finding that the expression of two genes [i.e., *flavonol synthase/flavanone 3-hydroxylase-like* (TDF #157-6) and *flavanone 3 hydroxylase-like protein* (TDF #134-14)] involved in flavonoid biosynthesis was induced by Al-toxicity except for similar root expression level of gene encoding lavanone 3 hydroxylase-like protein between two Al-treatments under 20 μM B (Table 1). This indicated that Al-toxicity might upregulate root biosynthesis of flavonoids, thus enhancing plant Al-tolerance. However, B-induced alleviation of Al-toxicity could not be explained by this way, because the expression levels of the two genes in Al-treated roots were not higher under 20 μM B than under 2.5 μM B (Table 1).

Four differentially expressed TDFs (i.e., TDFs #149-2, 216-2, 250-3 and 51-12) involved in lipid metabolism were isolated from roots (Table 1), demonstrating that B and Al interactions might alter root lipid metabolism. Carboxylesterases, which hydrolyze esters of short-chain fatty acids, play roles in plant defense, development, and secondary metabolism [43]. Our results showed that root expression of *probable carboxylesterase 12-like* (TDF #149-2) and *carboxylesterase 1-like* (TDF #216-2) kept unchanged and decreased in response to Al-toxicity under 2.5 μM B, respectively, but increased under 20 μM B, and that their expression level in Al-treated roots were higher under 20 μM B than under 2.5 μM B (TDF #149-2) or similar between the two B-treatments (TDF #216-2) depending on TDFs. The acylation of sterols has been thought to play a key role in maintaining free sterol homeostasis in the cell membranes. In *Arabidopsis*, sterol ester formation is catalyzed by phospholipid:sterol acyltransferase (PSAT), which displays homology with the mammalian lecithin-cholesterol acyltransferase (LCAT) [44]. Bouvier-Navé et al. [45] showed that the concentration of sterol esters decreased in leaves of *Arabidopsis psat1* mutants accompanied by an early leaf senescence phenotype, demonstrating the involvement of *PSAT1* in plant sterol homeostasis and leaf senescence. We found that root expression of gene encoding lecithin-cholesterol acyltransferase-like 4-like (TDF #250-3) in Al-treated roots decreased under 2.5 μM B, and kept unchanged under 20 μM B, and that its expression level in +Al roots was higher under 20 μM B than under 2.5 μM B (Table 1). The observed higher expression levels of genes encoding carboxylesterases and lecithin-cholesterol acyltransferase-like 4-like in 20 μM B + 1.2 mM Al-treated roots might contribute to the Al-tolerance of plants grown under 20 μM B.

As shown in Table 1, 10 TDFs (i.e., TDFs #136-3, 141-5, 87-2, 138-5, 138-3, 134-12, 18-2, 178-4, 54-2 and 80-2) related to amino acid and protein metabolism was altered by B and Al interactions. Adenosylhomocysteinase, which catalyzes the reversible hydrolysis of S-adenosyl-L-homocysteine (SAH, a strong inhibitor of transmethylation) to adenosine and L-homocysteine, is essential for maintaining the methyl cycling by the removal of SAH [46]. Zhao et al. [47] showed that 0.005 mM sodium nitroprusside (SNP) ameliorated Cd-induced toxicity in rice (*Oryza sativa*) and increased the abundance of adenosylhomocysteinase-like in Cd-treated rice roots. Our results showed that root expression of *adenosylhomocysteinase-like* (TDF #136-3) remained unchanged in response to Al-toxicity under 2.5 μM B and greatly increased under 20 μM B, and that its expression level in +Al roots was higher under 20 μM B than under 2.5 μM B (Table 1). Thus, adenosylhomocysteinase-like might play a role in B-induced alleviation of Al-toxicity. In addition, B and Al interactions also affected root expression of gene encoding S-adenosylmethionine-dependent methyltransferase At5g37990-like (TDF #141-5), which is involved in a variety of methylation reactions, and of gene encoding phosphomethylpyrimidine synthase (TDF, #87-2), which catalyzes the synthesis of 4-amino-2-methyl-5-phosphomethylpyrimidine from aminoimidazole ribotide in a radical S-adenosyl-L-methionine-dependent reaction (Table 1).

Nicotianamine (NA) aminotransferase (NAAT) plays a key role in the synthesis of mugineic acid family phytosiderophores (MAs) in graminaceous plants through catalyzing the amino group transfer of NA [48]. Takahashi et al. [49] showed that introduction of the barley NAAT gene into the nongraminaceous plant tobacco (*Nicotiana tabacum*), which produces NA but not phytosiderophores, caused a shortage of NA and decreases in the concentrations of Cu, Fe and Zn in leaves and floral organs of transgenic plants, indicating a role for NA in long-distance translocation of these metals. The Al-induced upregulation of root gene encoding nicotianamine aminotransferase A-like isoform X3 (TDF #138-5, Table 1) might contribute to Al-tolerance of plants by reducing Al concentration in stems and leaves. However, increased biosynthesis of NA in *Arabidopsis* and tobacco enhanced the tolerance of plants to high levels of metals [50].

LL-diaminopimelate aminotransferase is an enzyme involved in *meso*-diaminopimelate, a precursor of cell wall peptidoglycan and l-lysine in plants [51]. Tyrosine transaminase (also known as tyrosine aminotransferase) catalyzes the conversion of tyrosine to 4-hydroxyphenylpyruvic acid, a precursor for homogenetisic acid, plastoquinones and tocopherols, the latter of which function as radical scavengers and protect the plants against various stresses [52]. In this study, we first observed that root expression levels of genes encoding LL-diaminopimelate aminotransferase (TDF #138-3) and tyrosine transaminase family protein (TDF #134-12) kept unchanged in response to Al-toxicity under 2.5 μM B and decreased by Al-toxicity under 20 μM B (Table 1). It is unclear whether the two genes play a role in B-induced alleviation of Al-toxicity. Further studies are needed to answer this question.

Thiosulfate sulfurtransferase, which catalyzes the cyanide-dependent cleavage of thiosulfate to form thiocyanate and sulfite, is involved in sulfur metabolism, removal of cyanide, regulation of redox homeostasis, protection against biotic and abiotic stresses [53]. In this study, we observed that root expression of *thiosulfate sulfurtransferase 18-like isoform X1* (TDFs #18-2 and 178-4) decreased in response to Al-toxicity under 2.5 μM B, but increased under 20 μM B (Table 1). This implied that the gene might be involved in B-induced alleviation of Al-toxicity.

As shown in Table 1, five TDFs (i.e., TDFs #201-1, 25-4, 29-2,134-9 and 51-9) related to energy and carbohydrate metabolism were altered by B and Al interactions. Onda et al. [54] proposed that the interaction of root ferredoxin (Fd)-NADP reductase (FNR) with FD III played a key role in the efficient electron allocations from NADPH to Fd-dependent metabolism in root plastids. We found that root expression of gene encoding FNR, root isozyme 2 (TDF #201-1) upregulated in response to Al-toxicity under 20 μM B, which might be an adaptive response of plants to Al-toxicity. However, the abundance of FNR in *Lotus corniculatus* roots decreased in response to Al-toxicity [55].

### Genes related to stress response

Al-induced overproduction of reactive oxygen species (ROS) and lipid peroxidation have been observed in the roots of many plants including triticale [56], potato (*Solanum tuberosum*) [57], wheat [58], *Plantago algarbiensis* [59] and soybean [60]. To cope with the oxidative damage, plant cells are equipped with a scavenging system composed of antioxidants and antioxidant enzymes. Al-induced increases in both protein levels (activities) and expression levels of antioxidant enzyme genes have been reported in the roots of rice [61], triticale [56] and wheat [25]. Xu et al. [58] showed that Al treatment increased root activities of antioxidant enzymes, as well as the concentrations of antioxidants [i.e., AsA and reduced glutathione (GSH)] in two wheat genotypes: Yangmai-5 (Al-sensitive) and Jian-864 (Al-tolerant), and that Al-treated Jian-864 root tips had higher total antioxidant capacity and lower lipid peroxidation compared with Yangmai-5. They proposed that the total antioxidant capacity might play an important role in wheat plant Al-tolerance. Although the expression levels of *glutathione reductase* (GR) and *cytosolic-like and glutathione peroxidase 6* (TDFs #164-1 and 217-2) did not differ between 2.5 and 20 μM B-treated roots under Al-stress, the mRNA level of gene encoding glutathione S-transferase (GST) zeta class-like isoform X1 (TDF #78-4) in +Al roots was higher under 20 μM B than under 2.5 μM B (Table 1). Houde and Diallo [25] observed that GST expression level was higher in Al-tolerant than Al-sensitive wheat roots, concluding that GST might play a role in the detoxification of Al and ROS. Ezaki et al. [62] showed that overexpression of *GST* in transgenic *Arabidopsis* plants conferred tolerance to both Al and oxidative stresses. Thus, the observed higher expression level of *GST* in +Al roots under 20 μM B compared with under 2.5 μM B might enhance the tolerance of plants to Al.

Thioredoxins (Trxs) play a key role in redox balance regulation through thiol-disulfide exchange reactions [63]. Zhang et al. [64] found that transgenic rice plants overexpressing *OsTRXh1* (a subgroup I h-type Trx in rice) accumulated less H_2_O_2_ under salt stress, whereas more H_2_O_2_ was accumulated in the extracellular space of *OsTRXh1* knockdown plants compared with wild-type plants, demonstrating that *OsTRXh1* might play an important role in Trx-associated redox state regulation and plant stress responses. Lemaire et al. [65] showed that the expression of *Trxs m* and *h* in *Chlamydomonas reinhardtii* cells was induced by heavy metals such as Cd and Hg, concluding that Trxs was involved in defense mechanisms against heavy metals. Our results showed that the expression of *Trx m-type 4* (TDF #60-1) was induced by Al-toxicity only in 20 μM B-treated roots (Table 1), suggesting that *Trx m-type 4* might play a role in enhancing Al-tolerance by alleviating Al-induced oxidative stress under 20 μM B.

2-Alkenal reductase (AER) catalyzes the reduction of the α,β-unsaturated bond of 2-alkenals to produce *n*-alkanals. Transgenic tobacco plants overexpressing *Arabidopsis AER* displayed improved tolerance to photooxidative stress [66]. Recently, Yin et al. [67] showed that the suppression of lipid peroxide-derived aldehydes by AER provided an efficient defense mechanism against Al-toxicity. Thus, the Al-induced increase in root expression level of gene encoding 2-alkenal reductase (NADP^+^- dependent)-like (TDF #243-1, Table 1) might contribute to plant Al-tolerance by the detoxification of reactive carbonyls.

Heat shock proteins (HSPs)/chaperones have been known to play a key role in protecting plants against stress. Our results showed that root expression of gene encoding putative chaperone DnaJ-domain superfamily protein (TDF #178-1) downregulated in response to Al-toxicity under 2.5 μM B, and did not change under 20 μM B, and its expression level in +Al roots was higher under 20 μM B than under 2.5 μM B (Table 1), indicating that chaperones might play a role in B-induced alleviation of Al-toxicity.

Al-toxicity inhibits root growth by damaging the roots functionally and structurally, which consequently decreases water uptake, eventually resulting in dehydration stress in plant roots [40]. Consequently, the expression of some dehydration stress-related genes might be induced in Al-treated roots. As expected, root *dehydration responsive protein* (TDF #83-5) was strongly induced by Al-toxicity regardless of B concentration in the nutrient solution (Table 1). In addition, root expression level of gene encoding adenine nucleotide alpha hydrolases-like superfamily protein (TDF #219-3), a universal stress protein-like, was upregulated by Al-toxicity (Table 1). These data indicated that the two genes might play a role in plant Al-tolerance.

To conclude, our data demonstrated that in addition to enhancing the total ability to scavenge ROS, other mechanisms (i.e., ARE and chaperone DnaJ-domain superfamily protein) might be involved in B-induced alleviation of Al-toxicity.

### Genes related to autophagy and senescence

Autophagy is a process of self-degradation of cellular components including protein and organelle in a molecule degradation process in which cells recycle cytoplasmic nutrients and other cellular components when under stress conditions or during developmental transitions. This process can help plants to adapt the changing environment [68]. *RNAi-AtATG18a* transgenic *Arabidopsis* plants usually senesce earlier and are more sensitive to a variety of stressful conditions such as drought, salt and oxidative stresses compared with wild-type plants [68,69]. The observed lower expression level of *autophagy 18H-like protein* (TDF #158-1, Table 1) implied that root autophagy might be damaged by Al-toxicity, hence lowering plant Al-tolerance. However, B-induced alleviation of Al-toxicity can not be explained in this way, because the gene expression level in Al-treated roots kept unchanged regardless of B concentration in the nutrient solution (Table 1).

Senescence is a form of programmed cell death (PCD) and many senescence-associated genes (SAGs) have been identified in plants [70]. Al-toxicity results in premature cell maturation and senescence in plants [71]. Zhan et al. [72] showed that Al-induced PCD was promoted by *AhSAG*, a senescence-associated gene in peanut (*Arachis hypoganea*). Transgenic tobacco plants overexpressing *AhSAG* displayed lower ability of Al-tolerance than in antisense transgenic plants. In this study, we isolated nine differentially expressed TDFs encoding putative senescence-associated proteins (i.e., TDFs #2-1, 5-3, 139-8, 156-3, 141-7, 209-1, 217-1, 219-2 and 223-1). Their expression levels increased, decreased or kept unchanged in response to Al-toxicity depending on B concentration (Table 1), indicating that the whole progression of senescence in +Al roots was disturbed.

Protein degradation is the main biochemical process that occurs during plant senescence. Senescence associated proteases not only are involved in nutrient recycling, but also are involved in the regulation of the senescence process [73]. Differentially expressed *SAGs* isolated in our study, which participate in cellular protein degradation processes, included: *cysteine proteinase 15A-like* (TDF #176-9), *aspartic proteinase-like protein 1-like* (TDF #246-9), *serine-type peptidase* (TDF #87-3), *ubiquitin carboxyl-terminal hydrolase 22-like* (TDF #179-4) and *ubiquitin receptor RAD23c-like* (TDF #78-2). Root expression levels of these genes decreased or did not significantly change in response to Al-toxicity regardless of B concentration in the nutrient solution except that *ubiquitin carboxyl-terminal hydrolase 22-like* expression in 20 μM B-treated roots was upregulated by Al-toxicity (Table 1). These data also support above inference that the whole progression of senescence in +Al roots was disturbed.

### Genes related to signal transduction and hormone

Calmodulin, together with other calcium (Ca)-binding proteins, has been suggested to participate in heavy metal signaling by binding to Ca^2+^ [74]. Transgenic tobacco plants expressing a calmodulin-binding tobacco plasma membrane protein gene (designated NtCBP4, for *N*. *tabacum* calmodulin-binding protein) displayed enhanced Ni tolerance [75]. Okekeogbu et al. [76] observed that several Ca-binding proteins were induced in Al-treated tomato (*Solanum lycopersicum*) radicles, concluding that Ca-binding proteins might play a role in enhancing tomato plant tolerance to the secondary cellular stresses induced by Al-stress. Generally speaking, root expression levels of *putative Ca-binding protein CML19-like* (TDFs #19-4 and 19-5) were higher under 20 μM B than under 2.5 μM B regardless of Al concentration in the nutrient solution. This might related to the fact that the ameliorative effect of 20 μM B was better than that of 2.5 μM B.

Protein phosphorylation, a versatile post-translational modification (PTM), is involved in response to various environmental stresses including heavy metals (i.e., Mn, Cu, Cd and Al) [38,74,76,77]. Jonak et al. [77] showed that different kinase belonging to the MAPK family in alfalfa roots were induced by excessive Cd and Cu. Okekeogbu et al. [76] reported that MAPK was strongly induced in Al-treated tomato radicles. Zhou et al. [38] observed that Mn-toxicity decreased the expression levels of genes associated with phosphorylation except for enhanced expression of a *MAPK 1* gene in *C*. *grandis* leaves. Our results showed that all these differentially expressed genes [i.e., *protein kinase 2B* (TDF #89-2), *MAPK* (TDF #25-3), *probable receptor-like protein kinase At5g47070-like isoform X1* (TDF #140-2) and *SRSF protein kinase 1-like isoform 1* (TDF #246-3)] involved in phosphorylation were downregulated or not significantly affected by Al-toxicity depending on B supply and the kinds of protein kinase. Thus, phosphorylation of some proteins might be impaired in +Al roots. Like protein kinase, the transcript level of a gene [i.e., *protein phopsphatase 2C* (PP2C, TDF #51-15)] involved in dephosphorylation decreased or did not change in response to Al-toxicity depending on B supply (Table 1). This agrees with our previous report that the expression of *putative protein phosphatase 2a*, *regulatory subunit* was downregulated by Mn-toxicity in *C*. *grandis* leaves [38].

Tetraspanins, also called tetraspans or the transmembrane 4 superfamily (TM4SF), contain four transmembrane domains linked by a small outer loop (EC1), a larger outer loop (EC2) and a small inner loop (IL) and are involved in signaling pathways [78,79]. Root expression level of *tetraspanins-8-like* did not differ among B and Al combination except for a significant increase under 2.5 μM B + 0 mM Al (Table 1).

COP9 signalosome (CSN) complex, composing of eight subunits named CSN1 to CSN8 according to protein size, plays a role in diverse plant signaling pathways and developmental processes through regulating protein ubiquitination and degradation [80,81]. For example, RNA silencing of the *Arabidopsis* CSN5 subunit led to decreased auxin signaling. Gusmaroli et al. [80] observed that mutations in *CSN5A* caused a pleiotropic dominant negative phenotype, concluding that CSN^CSN5A^ was the major player in the derubylation of *Arabidopsis* Cullin1. As shown in Table 1, the expression of *COP9 signalosome complex subunit 5A* (CSN5A; TDF #138-6) was upregulated in -Al roots and downregulated in +Al roots by 20 μM B, respectively, and was enhanced in 2.5 μM B-treated roots and decreased in 20 μM B-treated roots by Al. Okekeogbu et al. [76] observed that the abundance of CSN6 was enhanced in Al-treated radicles of seeds derived from Al-treated tomato plants.

Ankyrin repeat-containing proteins, one of the most protein sequence motifs, play a role in cytoskeleton interactions, mitochondrial, toxins or signal transduction by mediating protein-protein interactions [82]. Shen et al. [83] observed that ankyrin repeat-containing protein 2A (AKR2A) played a key role in the biogenesis of *A*. *thaliana* ascorbate peroxidase 3 (APX3) by binding specifically to a sequence in APX3 (i.e., a transmembrane domain plus a few basic amino acid residues), concluding that AKR2A was an essential molecular for peroxisomal membrane-bound APX3. Our results showed that the expression of AKR At3g12360-like gene was higher in roots treated with 2.5 μM B + 0 mM Al than in other roots (Table 1), meaning that +Al roots might have lower or similar APX activity compared with -Al roots depending on B supply. This disagrees with the previous reports that the abundance of APX in wheat roots [84] and the activities of APX in *Allium cepa* roots [85] and ‘Cleopatra’ tangerine (*Citrus reshni*) leaves [86] increased in response to Al-toxicity. The difference between the expression level of APX gene and its activity (protein level) in response to Al might be due to PTMs.

WD (also known as Trp-Asp or WD40 or β-transducin) motifs are characterized by a conserved core of 40–60 amino acids, which usually form a tertiary propeller structure. WD repeat-containing proteins participate in a variety of cellular processes including signal transduction, vesicular trafficking, transcriptional regulation, apoptosis, cytoskeletal dynamics, ribosomal RNA biogenesis, and cell cycle control [87–90]. Mishra et al. [91] found that a *SiWD40* identified from foxtail millet, whose promoter interacted with the dehydration response element, was induced by various stresses such as salinity, dehydration and ABA, concluding that WD40 proteins might play a role in stress tolerance of foxtail millet. Lee et al. [92] demonstrated that a WD40 protein from *Brassica napus* might play a role in salt stress through ABA-dependent and/or -independent signaling pathways. Thus, Al-induced upregulation of *WD repeat-containing protein 26-like isoform X1* (TDF #87-7) in 2.5 μM B-treated roots might be involved in Al-tolerance.

Hormones are involved in plant Al-toxicity [93–95]. As shown in Table 1, *IAA-amino acid hydrolase ILR1-like 4-like* (TDF #178-5) expression was detected only in 20 μM B + 1.2 mM Al-treated roots. Chen et al. [96] reported that IAA-amino acid hydrolase ILR1-like 3 was induced in Hg-stressed rice roots. IAA-amino acid hydrolase ILR1, which was initially isolated in A. thaliana, releases active IAA from conjugates through cleaving IAA-amino acid conjugates [97]. Thus, free IAA level might be enhanced in 20 μM B + 1.2 mM Al-treated roots. This agrees with the report that Al treatments led to accumulation of endogenous IAA in wheat roots [96]. Zhou et al. [95] observed that IAA level increased in the base of the root and decreased in the root tips of 100 μM Al-treated alfalfa. Agami and Mohamed [98] reported that IAA pretreatment alleviated Cd-toxicity in wheat seedlings through enhancing the activities of antioxidant enzymes. Therefore, Al-induced expression of *IAA-amino acid hydrolase ILR1-like 4-like* in 20 μM B-treated roots might be an adaptive response of *C*. *grandis* plants to Al-toxicity. In addition, Yang et al. [94] showed that IAA increased the Al-induced secretion of malic acid anions from wheat roots. However, Al-induced secretion of malate and citrate did not differ between 2.5 and 20 μM B-treated *C*. *grandis* roots (Fig. 2).

In conclusion, signal transduction and hormone metabolism might be involved in B-induced alleviation of Al-toxicity.

### Genes related to gene regulation

As shown in Table 1, 15 TDFs (i.e., TDFs #188-3, 23-1, 138-4, 139-1, 177-3, 27-4, 132-1, 219-4, 177-8, 134-13, 134-4, 246-2, 83-2, 162-5 and 246-5) related to transcriptional regulation was altered by B and Al interactions. Al-induced changes in proteins and genes involved in gene regulation have also been observed in roots of soybean [99] and *Arabidopsis* [100].

Plant heat stress transcription factors (Hsfs), which are modular transcription factors, are involved in protective responses to various environmental stresses such as heat [101], heavy metals [102,103], and oxidative stress [102]. Shim et al. [103] showed that two orthologs of the plant class A4 Hsfs conferred Cd-tolerance in wheat and rice by enhancing the expression of Cd-tolerance gene, metallothionein. Using a dominant-negative approach, Davletova et al. [104] demonstrated that Hsfs were important sensors for H_2_O_2_ and were required at a relatively early stage of the oxidative stress acclimation response. Our results showed that Al treatment led to increased expression of *heat shock factor protein HSF24-lik*e (TDF #23-1) in 2.5 μM B-treated roots (Table 1). This indicated that Hsfs might play a role in the tolerance of plants to Al-toxicity. However, this could not explain why the ameliorative effect of 20 μM B was better than that of 2.5 μM B, because the gene expression level did not differ among roots treated with 2.5 μM B + 1.2 mM Al, 20 μM B + 1.2 mM Al, and 20 μM B + 0 mM Al (Table 1). In addition, the expression level of HSF domain class transcription factor (TDF #188-3) did not differ among four B and Al combinations except for a significant decrease under 20 μM B + 0 mM Al (Table 1). It appears that the response of Hsfs to Al-toxicity depends on B supply and Hsf member.

Pentatricopeptide repeat (PPR) proteins are required for a variety of post-transcriptional processes including RNA editing, RNA splicing, RNA cleavage and translation in plant organelles. Disruption of genes encoding PPR proteins often leads to severe phenotypes [105,106]. Our results showed that root expression of gene encoding putative pentatricopeptide repeat-containing protein At2g01510-like (TDF #139-1) decreased in response to Al under 2.5 μM B, and increased under 20 μM B, and that its expression level in +Al roots was higher under 20 μM B than under 2.5 μM B (Table 1), which might contribute to the tolerance of 20 μM B-treated plants to Al-toxicity. However, the expression of *pentatricopeptide repeat-containing protein* (TDF #177-3) was detected only in 2.5 μM B + 1.2 mM Al-treated roots (Table 1).

DNA-directed RNA polymerases catalyze the transcription of DNA into RNA. Our results showed that root expression of gene encoding DNA-directed RNA polymerase II subunit 1-like isoform X3 (TDF #27-4) was strongly downregulated by Al under 2.5 μM B and was not significantly affected under 20 μM B (Table 1), meaning that root transcription might be impaired by Al under 2.5 μM B, hence lowering the Al-tolerance of plants.

Al-toxicity leads to a degradation of DNA molecules and an apoptosis-like cell death in plant roots [85,107]. Shaked et al. [108] demonstrated the role of At5g63950/CHR24, a RAD26-like gene, in *Arabidopsis* DNA damage response and recombination. Our results showed that the expression of gene encoding DNA repair and recombination protein RAD26-like isoform X3 (TDF #132-1) was detected only in 20 μM B + 1.2 mM Al-treated roots (Table 1), which might contribute to the Al-tolerance of plants grown under 20 μM B. However, root expression of gene encoding DNA excision repair protein ERCC-1-like isoform X1 (TDF #219-4) was induced by Al-toxicity only under 2.5 μM B (Table 1).

Gag-Pol polyprotein is cleaved by proteases into functional peptides, which have been suggested to be essential for basic replication [109]. Our results showed that root expression of *Gag-pol polyprotein* (TDF #134-13) increased in response to Al under 20 μM B, and decreased under 2.5 μM B, and that its expression level in +Al roots was higher under 20 μM B than under 2.5 μM B (Table 1). This implied that *Gag-pol polyprotein* might be involved in B-induced alleviation of Al-toxicity.

### Genes related to cell transport

Twelve TDFs (i.e., TDFs #175-7, 59-3, 252-1, 141-8, 177-6, 134-5, 19-3, 180-2, 162-4, 78-3, 136-8 and 87-5) associated with cell transport in roots were altered by B and Al interactions (Table 1). Plant non-specific lipid transfer proteins (nsLTPs) are termed some LTPs which participate in the transfer of a broad range of lipids between membranes. Plant nsLTPs have been shown to play a role in mediating phospholipid transfer and the adaptation of plants to various environmental conditions [110]. Previous studies showed that root expression level of *nsLTP* (E30131) increased in response to Al-toxicity in Al-tolerant rice cultivar (Azucena), and decreased in Al-sensitive one (IR1552) [111], and that root mRNA level of *LTPs* was higher in Al-tolerant than in Al-sensitive soybean genotype [21]. The major facilitator superfamily (MFS), a class of membrane transport proteins, plays a role in plant metal homeostasis [112]. Haydon and Cobbett [113] showed that an *Arabidopsis* MFS member, Zinc-Induced Facilitator 1 (ZIF1) localized at the tonoplast, was involved in Zn-tolerance, demonstrating that MFS transporters might inﬂuence plant ion homeostasis. In addition, plant MFS transporters, which belong to the Pht1 and Pht4 families, regulate high- and low-affinity inorganic phosphate transport, respectively [114,115]. Our results showed that genes encoding non-specific lipid-transfer protein-like protein At2g13820-like (TDF #175-7) and putative MFS protein (TDF #59-3) were expressed only in 20 μM B-treated roots (Table 1), suggesting that the two genes might play a role in B-induced alleviation of Al-toxicity.

Citrate binding protein (CBP) is involved in plant vacuolar citrate transport [116]. Our finding that root expression level of citrate-binding protein-like gene (TDF #252-1) increased in response to Al-toxicity (Table 1) agrees with our results that Al-toxicity induced the secretion of citrate from roots (Fig. 2A). Interestingly, Al-induced upregulation of citrate-binding protein-like gene was lower under 20 μM B than under 2.5 μM B, which could be due to the amelioration of Al-toxicity by B.

Membrane traffic is required for normal cellular function by which molecules are transported between organelles in the post-Golgi network [117]. Peiter et al. [118] proposed a mechanism for metal tolerance involving membrane trafficking. Our results showed that root expression levels of genes encoding patellin-2-like, membrane lipoprotein, ADP-ribosylation factor GTPase-activating protein AGD3-like, Ras-related protein RABA1f-like and protein transport protein Sec61 subunit alpha-like (TDFs #141-8, 177-6, 162-4, 134-5 and 19-3) increased or kept unchanged in response to Al toxicity depending on B concentration in the nutrient solution (Table 1), indicating that the membrane traffic might be enhanced in Al-treated roots, thus conferring plant Al-tolerance. However, root expression levels of genes encoding syntaxin-71-like, putative clathrin assembly protein and target of Myb protein 1-like isoform X1 (TDFs #180-2, 78-3 and 136-8) decreased or did not change in response to Al-toxicity (Table 1).

### Genes related to cell wall modification

Cell wall has been considered as the major site of Al-toxicity [119]. As expected, four TDFs (i.e. 124-1, 17-1, 51-1 and 148-1) involved in cell wall modification in roots were altered by B and Al interactions (Table 1). Our results showed that root expression of gene encoding putative glycosyl hydrolase family 10 protein (TDF #51-1), a family of glycoside hydrolases, decreased in response to Al-toxicity under 2.5 μM B and increased under 20 μM B, and that its expression level in +Al roots was higher under 20 μM B than under 2.5 μM B (Table 1). Duressa et al. [21] showed that the expression level of gene encoding glycosyl hydrolase family 3 protein/o-glycosyl cpds was higher in Al-tolerant than in Al-sensitive soybean genotype. Thus, glycosyl hydrolase might be involved in B-induced alleviation of Al-toxicity.

Pectate lyases degrade plant cell walls, causing tissue maceration and death [120]. We found that the expression of *probable pectate lyase 8-like* (TDF #124-1) in roots was down-regulated by Al-toxicity (Table 1), as previously obtained on Al-treated roots of aspen (*Populus tremula*) [24], indicating that pectate lyases might play a role in plant Al-tolerance. By contrast, the expression of gene encoding probable pectinesterase/pectinesterase inhibitor 61-like (TDF #17-1) was detected only in 2.5 μM B + 1.2 mM Al-treated roots (Table 1), which disagrees with the previous report that Al downregulated alfalfa root expression of pectinesterase inhibitor gene [22].

In conclusion, we demonstrated the alleviation of Al-toxicity by B in *C*. *grandis* seedlings. The alleviation might be associated with relatively little Al transport from roots to leaves (shoots) rather than through increasing B concentration in roots and leaves, because its concentration was higher in +Al roots and leaves than in -Al ones. The molecular mechanisms underlying these processes are only beginning to understand. In this study, we first used the cDNA-AFLP to investigate the gene expression patterns in *C*. *grandis* roots in response to B and Al interactions, and successfully isolated 100 differentially expressed TDFs including some novel B-Al interaction responsive genes. B appears to alleviate Al-toxicity in *C*. *grandis* roots by the following several aspects: (*a*) improving the total ability to scavenge ROS and aldehydes; (*b*) increasing the expression levels of genes related to lipid (i.e., *carboxylesterases and lecithin-cholesterol acyltransferase-like 4-like*), amino acid (i.e., *nicotianamine aminotransferase A-like isoform X3*), S (i.e., *thiosulfate sulfurtransferase 18-like isoform X1*) and energy (*i*.*e*., FNR, root isozyme 2) metabolisms; and (*c*) upregulating gene expression related to cell transport (i.e., *non-specific lipid-transfer protein-like protein At2g13820-like* and *MFS protein*). In addition, genes related to Ca signal and hormone, gene regulation, and cell wall modification might also play a role in B-induced alleviation of Al-toxicity. Therefore, our study reveals some novel evidence for the B-induced alleviation of Al-toxicity at the transcriptional level, and increases our understanding of the molecular mechanisms on B-induced alleviation of Al-toxicity. Our results also are useful to us for obtaining the key genes responsible for plant Al-tolerance.

## Supporting Information

S1 Fig A representative picture of a silver-stained cDNA-AFLP gel showing the differentially expressed TDFs in C. grandis roots in response to B and Al interactions using one EcoR I selective primer (EcoR I-AG) and nine Mes I selective primers (Mes I-CC, CG, CT, CA, GC, GG, GT, GA and TC). 1: 2.5 μM B + 0 mM Al; 2: 2.5 μM B + 1.2 mM Al; 3: 20 μM B + 0 mM Al; 4: 20 μM B + 1.2 mM Al. Arrows indicate differentially expressed TDFs.
(DOC)

S2 Fig Correlation analysis of qRT-PCR results and cDNA-AFLP data for selected genes. Gene encoding 5'-3' exoribonuclease 3-like isoform X2 (TDF #162–5) was not included in the analysis because the TDF was detected only in 2.5 μM B + 1.2 mM Al-treated roots.
(DOC)

S1 Table Specific primer pairs used for qRT-PCR expression analysis. (DOC)
