# Supplementary material for: Mechanisms on Boron-Induced Alleviation of Aluminum-Toxicity in Citrus grandis Seedlings at a Transcriptional Level Revealed by cDNA-AFLP Analysis
Source: PLoS One. 2015 Mar 6;10(3):e0115485. doi: 10.1371/journal.pone.0115485 (PMC4352013; doi:10.1371/journal.pone.0115485)
Supplement: S1 Fig — 1: 2.5 μM B + 0 mM Al; 2: 2.5 μM B + 1.2 mM Al; 3: 20 μM B + 0 mM Al; 4: 20 μM B + 1.2 mM Al. Arrows indicate differentially expressed TDFs. (DOC) [file pone.0115485.s001.doc]

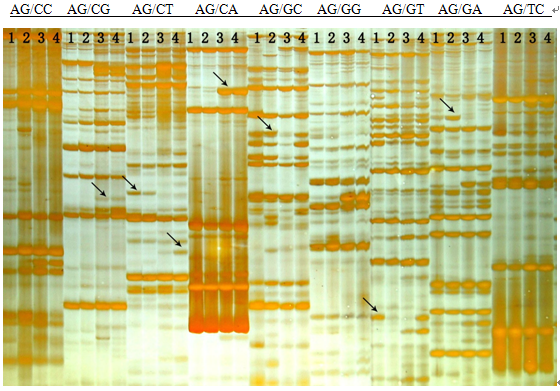


Figure S1: A representative picture of a silver-stained cDNA-AFLP gel showing the differentially expressed TDFs in *C. grandis* roots in response to B and Al interactions using one *Eco*R I selective primer (*Eco*R I-AG) and nine *Mes* I selective primers (*Mes* I-CC, CG, CT, CA, GC, GG, GT, GA and TC). 1: 2.5 μM B + 0 mM Al; 2: 2.5 μM B + 1.2 mM Al; 3: 20 μM B + 0 mM Al; 4: 20 μM B + 1.2 mM Al. Arrows indicate differentially expressed TDFs.
