# Supplementary material for: Mechanisms on Boron-Induced Alleviation of Aluminum-Toxicity in Citrus grandis Seedlings at a Transcriptional Level Revealed by cDNA-AFLP Analysis
Source: PLoS One. 2015 Mar 6;10(3):e0115485. doi: 10.1371/journal.pone.0115485 (PMC4352013; doi:10.1371/journal.pone.0115485)
Supplement: S2 Fig — Gene encoding 5'-3' exoribonuclease 3-like isoform X2 (TDF #162–5) was not included in the analysis because the TDF was detected only in 2.5 μM B + 1.2 mM Al-treated roots. (DOC) [file pone.0115485.s002.doc]

Figure S2: Correlation analysis of qRT-PCR results and cDNA-AFLP data for selected genes. Gene encoding 5'-3' exoribonuclease 3-like isoform X2 (TDF #162-5) was not included in the analysis because the TDF was detected only in 2.5 μM B + 1.2 mM Al-treated roots.
