# Supplementary material for: Mechanisms on Boron-Induced Alleviation of Aluminum-Toxicity in Citrus grandis Seedlings at a Transcriptional Level Revealed by cDNA-AFLP Analysis
Source: PLoS One. 2015 Mar 6;10(3):e0115485. doi: 10.1371/journal.pone.0115485 (PMC4352013; doi:10.1371/journal.pone.0115485)

Table S1: Specific primer pairs used for qRT-PCR expression analysis

| *Genes* | *Forward primers (5´→3´)* | *Reverse primers (5´→3´)* |
| --- | --- | --- |
| 19-3 | CAAAGTTGGAGGATTATGAG | TGATTGCTATCGGTGAGG |
| 54-2 | GCGATCCAATTCAAGGAG | TGAGTAACGCCAAGACCC |
| 60-1 | CGTGACTGCGATCCAATT | TGTGCGAGGCTCAAGAAA |
| 83-2 | CCGACTTCTGAAAGGGAT | CCGTACTCAGGACTCATCG |
| 83-5 | CGATCCAATTCCGCGTCC | GTCCTGAGTAAATGCGATG |
| 87-7 | ATTCCGATGAGTGACTGC | GCTTGTTCAAATCCACCA |
| 134-5 | ATTCGCCACTCGCAGCAT | TCGGGTGACATCATAGACAAGC |
| 157-6 | GAGCCCATTCTCCGTCTG | ATCACTACCCACCGTGCC |
| 162-5 | TATCCACCCAGACCTATG | TCCTGAGTAAAGCTACCAG |
| 178-4 | CAATTCGAGGAAGGTCAT | ACAGCCCACTACAAGACG |
| 219-2 | CAATGATAGGAAGAACCGAC | CGCTCAACGAGAACAGAA |
| 219-3 | AGTCCTGAGTAAGTGGGTGA | CCTCCAGCAAGGGTAAAT |
| 243-1 | TGAAATAGGTAAGCCCAAGA | TGCATCATCAAAGCCAAG |
| *Actin* | AGAACTATGAACTGCCTGATGGC | GCTTGGAGCAAGTGCTGTGATT |

Supplementary Figure 1. A representative picture of a silver-stained cDNA-AFLP gel showing the differentially expressed TDFs in *C. grandis* roots in response to B and Al interactions using one *Eco*R I selective primer (*Eco*R I-AG) and nine *Mes* I selective primers (*Mes* I-CC, CG, CT, CA, GC, GG, GT, GA and TC). 1: 2.5 μM B + 0 mM Al; 2: 2.5 μM B + 1.2 mM Al; 3: 20 μM B + 0 mM Al; 4: 20 μM B + 1.2 mM Al. Arrows indicate differentially expressed TDFs.


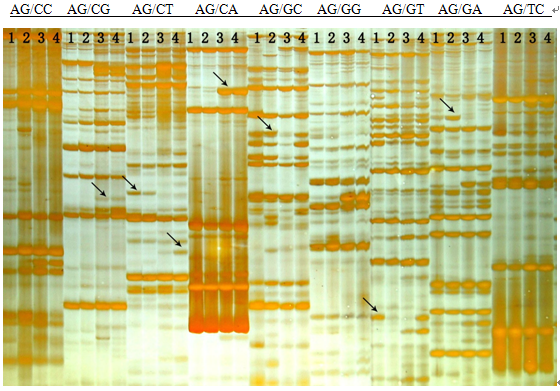

Supplement: S1 Table — (DOC) [file pone.0115485.s003.doc]
